# Women’s suggestions on how to improve the quality of maternal and newborn hospital care: a qualitative study in Italy using the WHO standards as framework for the analysis

**DOI:** 10.1186/s12884-020-02893-0

**Published:** 2020-04-06

**Authors:** Marzia Lazzerini, Chiara Semenzato, Jaspreet Kaur, Benedetta Covi, Giorgia Argentini

**Affiliations:** WHO Collaborating Centre for Maternal and Child Health, Institute for Maternal and Child Health IRCCS Burlo Garofolo, Via dell’Istria 65/1, 34137 Trieste, Italy

**Keywords:** Women, Newborn, Service users, Quality of care, Standards, WHO, Hospital, Qualitative study

## Abstract

**Background:**

A recent systematic review identified very few studies on women’s views on how to improve the quality of maternal and newborn care (QMNC). This study aimed at exploring the suggestions provided by women, after hospital delivery in Italy, on how to improve the QMNC.

**Methods:**

A questionnaire, containing open questions to capture suggestions on how to improve QMNC, was used to collect suggestions of mothers who gave birth a tertiary care referral hospital in Northeast Italy, between December 2016 and September 2018. Two authors independently used thematic analysis to analyse women’s comments, using the WHO Standards for improving the QMNC as framework for the analysis.

**Results:**

Overall 392 mothers provided a total of 966 comments on how to improve the QMNC. Overall 45 (11.5%) women made suggestions pertinent to “provision of care”, 222 (56.6%) to the “experience of care”, 217 (55.4%) to “physical or to human resources”. The top five suggestions were: 1) increase presence of a companion during the whole hospitalization (28.3% of women); 2) improve bathrooms and showers (18.4%); 3) improve effective communication from staff (14.0%); 4) improve staff professionalism, empathy, and kindness (13.5%); 5) increase support and information on how to provide care to the newborn (11.2%). Overall, 158 (16.4%) suggestions could not be classified in any WHO Standards, and among these most (72.1%) were related to physical structures, such as: decrease the number of patients per room; create areas for visitors; avoid case mixing in the same room; reduce rooming-in/better support the mother. Overall 62 (15.8%) women expressed appreciations.

**Conclusions:**

Collecting the women’s views on how to improve the QMNC after hospital delivery highlighted critical inputs on aspects of care that should be improved in the opinion of service-users. More investments should be made for establishing routine systems for monitoring patients experience of care. Data collected should be used to improve QMNC. WHO Standards may be further optimized by adding items emerging as relevant for women in high-income countries.

## Background

Health 2020, the World Health Organisation (WHO) policy framework and strategy for the European Region [1], identifies quality of maternal and newborn care (QMNC) as a key determinant of maternal and newborn health outcomes, of health services expenditures, and as a crucial aspect of human rights. The importance of QMNC - which includes the dimension of patient-centred care - is also recognised by many other policy documents, including the WHO Global Strategy for women and children, the agenda for Sustainable Development Goals (SDGs), and it is in general is widely accepted by many other groups and organizations [2–5]. In the recent years there has been an increasing recognition on the fact that cross the world many women experience low quality care, and often disrespectful, abusive, or neglectful treatment during childbirth in facilities [6–10]. The importance on providing a good “experience of care” during childbirth is now emphasized in many recent documents, such as the “WHO Guidelines of Intrapartum Care for a Positive Childbirth Experience” [11].

Despite high-income countries in the European Region, including Italy [6], are characterized by low maternal and newborn mortality when compared to resources settings, yet several challenges in the QMNC persist. Current evidence suggests that in many high-income countries within the European Region the implementation of good practices based on evidence is still unsatisfactory [12–17], with a diffuse tendency to overmedicalisation [12, 15–17], and, frequently, a culture of “paternalism”, with low participation of women in decision making [17]. In general, even in high-income countries, such as Italy mothers’ qualitative perception of the experience of care is very not included in the routine assessment of the QMNC, and not considered for health services planning purposes [6].

Among the many initiatives aiming at improving the QMNC, in 2015 the WHO developed a framework which defines clearly key components of quality services for the mother and the newborn [18]. The WHO framework identifies the following three key dimensions of the QMNC: 1) “provision of care”, including evidence-based practices, efficient information and referral systems; 2) “experience of care” including effective communication, respect, dignity and emotional support; 3) the availability of resources, including “competent, motivated human resources” and “appropriate physical resources” [18]. Based on these eight key dimensions of the framework [18], WHO developed eight Standards for improving the QMNC in health facilities”, further declined into 31 quality statements, and over 300 quality measures, in an attempt of defining what health care planners, managers and care providers should ensure to guarantee high-quality care around the time of birth [19]. The WHO Standards were developed based on the existing literature and through a large consultation with experts and represent a very comprehensive set of measures related to the QMNC [19]. However, as documented in a recent systematic review [20], what matters to women during childbirth is still relatively poorly documented. Specifically, among 35 studies identified by the systematic review [20], all had a very small sample (ie, maximum 35 women), with the only exception being a study in Sweden (908 women), one in Australia (202 women), and one in India (85 women) [21–23]. Additionally, these studies did not documented suggestions of women, collected after delivery, on how to improve QMNC, but rather expectations before delivery [21, 22] or changes in cultural belief through generations [23]. None study from Italy has ever reported women’s suggestions on how to improve the QMNC [20]. This qualitative study aimed at exploring the suggestions provided by women, after hospital delivery in Italy, on how to improve the QMNC.

## Methods

### Study design

This was a qualitative study, and in reporting it, we used the Standards for Reporting Qualitative Research [24]. (Supplementary Table 1).

### Setting

The study was conducted between December 2016 and September 2018 in a large public tertiary level university referral hospital in Northeast Italy, the Institute of Research for Maternal and Child Health Burlo Garofolo, Trieste. Every year about 1700–1800 mothers give birth in the hospital.

### Data collection

Mothers who gave birth in the hospital from December 2016 to September 2018 were invited to participate. Exclusion criteria were: maternal death, perinatal death (including stillbirth), refuse to participate, psychiatric or psychosocial problems with inability to fill in the questionnaire (as assessed by a psychiatrist or by a social assistant), age under 18 years old, and language barriers.

Data were collected using a field-tested, anonymous, self-administrated, questionnaire in the local language (Italian). The questionnaire and the procedure for its validation, together with preliminary results of a survey conducted have been reported in a previous publication [25]. Briefly, the questionnaire was developed after a large review and thematic analysis of other existing tools and reference standards [25]. The questionnaire was tested in a sample of voluntary mothers, with different characteristics (age, education, parity, etc) who reviewed the questionnaire individually and provided a written feedback [25]. The draft version of the questionnaire was also submitted to a panel of experts with experience on research in QMNC issues (obstetricians, neonatologists, midwives, epidemiologists), for content validity and construct coherence [25]. Experts reviewed the questionnaire independently in a firth phase and in an extensive group discussion meeting, in a second phase [25]. The questionnaire was optimized according to the feedback received, and the final version was re-tested in a second group of voluntary mothers [25]. The questionnaire included open questions to collect any type of suggestion, comment or request from women on how to improve the QMNC. Mothers could decide on a voluntary basis whether to fill these open questions. The questionnaire also collected socio-demographic information of women, and a question on women satisfaction with the care received, scored on a Likert scale from 1 (minimal) to 10 (maximal) satisfaction [25]..

The questionnaire and the overall objectives of the study were presented to the mothers in the post-delivery period, during their stay the post-delivery ward (usually less than 3 days after delivery), by a trained research midwife, not involved in case management. Mothers were enrolled from Monday to Saturday, and they could return the filled questionnaires directly to the operator, or in a dedicated box available in the ward 24/24 h and 7/7 days.

### Data analysis

Two authors independently created an Excel spreadsheet of all women’s comments, and used thematic analysis methods to conduct initial open coding on each relevant text unit [26]. The women’s comments were classified according to the WHO framework and Standards [18, 19]. The WHO framework and Standards [18, 19] include three main domains (“experience of care”, “provision of care” and “resources”), eight Standards and 31 Quality Statements. The eight WHO Standards were used as major themes, and the 31 Quality Statements were used as second level themes. Each theme was then further expanded, based on the themes emerging from women’s comments, to develop the final axial coding scheme. Axial coding is widely accepted in qualitative literature as a sufficient method to disaggregate core themes during qualitative analysis [26–28]. Two researchers applied independently the axial codes systematically to the data by hand-sorting the text units into themes and sub-themes. Any theme emerging from women’s comment and not included in the WHO standard was added as additional theme. We calculated the total number of comments and the frequency of comments in each theme, using two distinct denominators: number of women (*N* = 392); total comments (*N* = 966). If a comment pertained to more than one theme (eg, both to experience and provision of care), we opted for inputting it in both themes, in order to capture all relevant themes. If the content of one comment was unclear to both researchers, it was labelled as “unclear”. Comments without any specific suggestion were classified as follows: appreciations, negative comments, unclear. Any disagreements on thematic analysis was solved by discussion between the two authors and consensus sough through two senior authors. Results are reported in tables and text.

## Results

### Women’s characteristics

Overall 392 mothers provided 966 comments related on how to improve the QMNC. Characteristics of mothers are reported in Table 1. The median age was 33.5 years (range = 18–46) and 91.6% had an Italian nationality. More than half of mothers (56.5%) were primiparous, and nearly all (98.7%) had a single pregnancy. Over half (58.2%) were highly educated (Bachelor’s degree or specialist degree). In terms of key outcomes, overall 74 (18.9%) had an elective caesarean section, while 116 (29.6%) had an emergency caesarean section, while 43 (11.0%) had their baby in the intensive care unit. Most of mothers (68.1%) were highly satisfied with the care received, while only 40 (10.2%) were not satisfied. There were not significant differences between mothers who provided suggestion and those who did not, except for slightly more mother in the first group having a post-graduate education (19.3% vs 13.3%, *p* = 0.02 see Supplementary Table 2).

**Table 1:** Characteristics of mothers

	N (%) (***N*** = 392)
**Age, median (range)**	33.5 (18–46)
**Italian nationality**	360 (91.6)
**Primiparous**	222 (56.5)
**Multiple pregnancy**	5 (1.3)
**Education**	
No formal education	0 (0)
Primary school	1 (0.3)
Lower secondary education	23 (5.9)
Upper secondary education	138 (35.1)
Degree	153 (38.9)
Post-graduate studies	76 (19.3)
**Caesarian section**	
Elective cesarean section	40 (10.2)
Emergency cesarean section	55 (14.0)
**Baby in intensive care unit**	43 (11.0)
**Maternal satisfaction with the care received**^a^	
Not satisfied	40 (10.2)
Fairly Satisfied	83 (21.2)
Highly satisfied	267 (68.1)
Missing	2 (0.5)

^a^Maternal satisfaction was assessed on a Likert Scale of 1 (not at all satisfied) to 10 (maximum satisfaction). Women with a score 1 to 5 were considered “Not satisfied”. Women with a score of 6–7 were considered “Fairly satisfied”. Women with a score equal or above 8 were considered “Highly satisfied”

### Women’s suggestions on how to improve QMNC

Most women made more than one suggestion, with a mean rate of 2.5 suggestions per women (median 2.0, range 1 to 10) (Table 2). Overall 45 (11.5%) women made suggestions pertinent to provision of care, 222 (56.6%) to the experience of care, 217 (55.4%) to human and physical resources.

**Table 2:** Number of women’s suggestions by domain of quality of care

Domain of quality of care	On total women (***N*** = 392) ^a^	On total comments (***N*** = 966)
Provision	45 (11.5)	48 (5.0%)
Experience	222 (56.6)	316 (32.7%)
Human and physical resource	217 (55.4)	355 (36.7%)
Not included in the WHO Standards	136 (34.7)	158 (16.4%)
Not including a suggestion	89 (9.2%)	89 (9.2%)

^a^ Most women made more than one comment, therefore the total exceeds 100%

Overall, 158 (16.4%) suggestions could not be classified in any WHO Standards. In addition, 89 (9.2%) comments did not include any practical suggestion.

The top five women’s suggestions were: 1) increase presence of a companion during the whole hospitalization (28.3% of women); 2) improve bathrooms and showers (18.4%); 3) improve effective communication from staff (14.0%); 4) improve staff professionalism, empathy, and kindness (13.5%); 5) increase support and information on how to provide care to the newborn (11.2%). Detailed results are reported in the following paragraphs.

#### Provision of care

Among these 48 suggestions, half [24] were requests of improving counselling and support on breastfeeding, while about one third [17] were related to options of pain relief during labour and childbirth (Table 3). For example, a mother wrote “*There is need for more anaesthetists 24/24h for performing epidural: I had terrible pain and I felt that because of this my labour was not progressing”*.

**Table 3:** Women’s suggestions related to the provision of care

3rd level – WHO Standards	2nd level – Quality statements	1st level – Women’s suggestions	On total women (***N*** = 392)	On total comments (***N*** = 966)
Standard 1: every woman and newborn receive routine, evidenced-based care and management of complications during labour, childbirth, post -partum, according with WHO Guidelines	1.1a Timely appropriate care during labour and childbirth	Provide different options for pain relief during labour and childbirth	17 (4.3)	17 (1.8)
	1.1b Routine care for newborn immediately after birth	Encourage skin to skin contact for at least 1 h after birth	4 (1.0)	4 (0.4)
		Perform umbilical cord clamped after 1–3 min	1 (0.3)	1 (0.1)
	1.1c Routine postnatal care for mother and newborn	Improve breastfeeding counselling and support from a skilled health care provider	24 (6.1)	24 (2.5)
	1.2 Interventions for preclampsia/eclampsia according to WHO GL		0	0
	1.3 Interventions for PPH according to WHO GL		0	0
	1.4 Interventions for delay/obstructed labour according to WHO GL	Improve management of obstructed labour	1 (0.3)	1 (0.1)
	1.5 Newborns who are not breathing receive stimulation and resuscitation within 1 min after birth according to WHO GL		0	0
	1.6a Appropriate care for preterm and small babies according to WHO GL		0	0
	1.7a Interventions for women with or at risk of infection according to WHOGL		0	0
	1.7b Antibiotic treatment for newborns with suspected infection or risk factors according to WHO GL		0	0
	1.8 Precautions for preventing hospital-acquired infections		0	0
	1.9 No unnecessary or harmful practices during labour, childbirth, post-partum	Reduce medicalisation	1 (0.3)	1 (0.1)
Standard 2: the health information system enables use of data to ensure early, appropriate action to improve the care of every woman and newborn	2.1 Complete, accurate, standardized medical record		0	0
	2.2 Mechanism for data collection, analysis and feedback for monitoring and improving performance around childbirth		0	0
Standard 3: every woman and newborn with condition that cannot be dealt with effectively with the available resources is appropriately referred	3.1 Assessment to determine whether referral is required, and the decision to refer is made without delay		0	0
	3.2 If needed, the referral follows a pre-established plan that can be implemented without delay		0	0
	3.3 For every referral within or between health facilities: appropriate information exchange and feedback to relevant health care staff		0	0
Extra	Not included in WHO Standards	Increase access to labour/birth in water	7 (1.8)	7 (0.7)
		Increase access to home birth with skilled attendant, coordinated by the health facility	1 (0.3)	1 (0.1)
		Create perineal rehabilitation clinics	1 (0.3)	1 (0.1)
		Create a system for the mother to call for help from different type of staff when in bed during the post-delivery (ie, emergency button to call for midwives separate from emergency button for nurses)	1 (0.3)	1 (0.1)

*Abbreviations*: *GL* Guidelines, *PPH* Post-partum haemorrhage, *WHO* World Health Organization

There was a low number of suggestions related to the other WHO Quality measures of WHO Standard 1 (“Every woman and newborn receives routine, evidence-based care and management of complications during labour, childbirth, post-partum, according with WHO Guidelines”), and zero comments related to the WHO Standard 2 (“The health information system enables use of data to ensure early, appropriate action to improve the care of every women and newborn”) and the WHO Standard 3 (“Every woman and newborn with condition that cannot be dealt with effectively with the available resources is appropriately referred”).

Overall 10 women made suggestions that could not be classified in any WHO Standards, with the most frequent being improving access to labour/birth in water (1.8% of total women).

#### Experience of care

The domain of experience of care accounted for 316 (32.7%) of total comments, with over half of women (56.6%) providing suggestions (Table 2). Additionally, the top one most frequent women’s request among total comments pertained to this domain, namely: increase presence of a companion during the whole period of hospitalization (28.3% of women) (Table 4). For example, one mother wrote: “*father should not be treated as the other visitors; they should be allowed to live with us this experience, they should stay with us all the time they want and can*”, while one added “*more support from fathers, with more flexible access to the ward could help us to rest; we need it so much, and it is in the interest of the baby”.*

**Table 4:** Women’s suggestions related to experience of care

3rd level – WHO Standard	2nd level – Quality statement	1st level - Women’s suggestions	On total women (***N*** = 392)	On total comments (***N*** = 966)
Standard 4: Communication with women and their families is effective and responds to their needs and preferences	4.1 All women and families receive info about the care and have effective interactions with the staff	Improve communication with patients (ie, active listening, asking/responding to questions, verifying the understanding, supporting women in problem solving)	55 (14.0)	56 (5.8)
		Increase availability of easily understandable health education materials	25 (6.4)	25 (2.6)
		Improve empathic behaviours	4 (1.0)	4 (0.4)
	4.2 Coordinate care, with clear, accurate information exchange between relevant health and social care professionals	Strengthen coordinated care and communication among health professionals	30 (7.7)	30 (3.1)
		Improve effective handover at shift changes and information exchange among different health professionals	13 (3.3)	13 (1.3)
		Health professionals should introduce themselves	8 (2.0)	8 (0.8)
Standard 5: Women and newborn receive care with respect and preservation of their dignity	5.1 Privacy around labour and childbirth, confidentiality respected	Ensure privacy during examinations and treatment and confidential	3 (0.8)	3 (0.3)
	5.2 No mistreatment such as physical, sexual or verbal abuse, discrimination, neglect, detainment, extortion or denial of services	Improve respect and dignity of mothers	15 (3.8)	15 (1.6)
		The mothers of small, sick newborns should be able to stay close to their babies	9 (2.3)	9 (0.9)
	5.3 All women have informed choices in the services they receive, and the reasons for interventions or outcomes are clearly explained	Improve tools/procedures for administering informed consent to women before examinations and procedures	8 (2.0)	8 (0.8)
Standard 6: Every woman and her family are provided with emotional support that is sensitive to their needs and strengthens the woman’s capability	6.1 Every woman is offered the option to experience labour and childbirth with the companion of her choice	Allow more extended presence of a companion of choice during labour and childbirth (eg, free visiting hours for at least one person during the whole hospitalization)	111 (28.3)	111 (11.5)
	6.2 Every woman receives support to strengthens her capability during childbirth	Encourage more women to adopt the position of their choice during labour and to walk around freely	13 (3.3)	13 (1.3)
		Increase respect for women’s choice and preferences	21 (5.4)	21 (2.2)
Extra	Not included in WHO Standards	Consider reshaping visiting time and rooms for relatives according to mothers’ preferences	25 (6.4)	25 (2.6)
		Strengthen access to one to one care (ie, care by the same doctor one to one)	5 (1.3)	5 (0.5)
		Facilitate the co-existence of public and private care within the same facility	4 (1.0)	4 (0.4)

Overall, about one out of six (14.0%) of mothers highlighted the need for improving communication. For example, one mother wrote: “*more information is needed for the mother”*, while another added: *“we need to be listening to, we need doctors to be able to listen more to what are our needs”.*

Other frequent women’s requests were: strengthened coordinated care and improved communication among hospital staff (7.7%). For example, a mother wrote “*every staff says a different thing, and this is confusing, communication among staff should be improved”,* while another added: “*communication to patients need to be respectful, sometimes I felt treated as I was not able to understand nor to do nor to decide anything”.*

Overall 34 women’s requests in this area were not related to any WHO Standard, with the most frequent being the need of better regulating visiting times for relatives (6.4% of women).

#### Human and physical resources

The domain of human and physical resources accounted for 355 (36.7%) of total comments, with over half of women (55.4%) providing suggestions in this area (Table 2). Additionally, three of the top five most frequent women’s requests among total comments pertained to this area (Table 5): improve bathrooms and showers (18.4% of total women); improve staff professionalism, empathy, and kindness (13.5%); increase support and information on how to provide care to the newborn (11.2%). For example, one mother reported “*I had very different experience with two different midwives, while all staff should all be able to provide equally good care”*. Another added: *“nurses should understand that most of mothers are at the first breastfeeding experience; they should be more patient and collaborative, and less judgmental; also, they should give more consistent advices”*.

**Table 5:** Women’s suggestions related to physical structures and human resources

3rd level – WHO Standard	2nd level – Quality statement	1st level – Women’s suggestions	On total women (***N*** = 392)	On total comments (***N*** = 966)
Standard 7: for every woman and newborn, competent, motivated staff are consistently available to provide routine care and manage complications	7.1 Access at all times to at least one skilled birth attendant and support staff	More professional and dedicated care during labour and birth guaranteed at any time	23 (5.9)	23 (2.4)
		More professional attention/information and support after child birth, especially from newborn nurses, to cover all needs of the post-partum period, especially related to the newborn	44 (11.2)	44 (4.6)
		Improve availability of hospital staff (shall be available at all times in a sufficient number)	32 (8.2)	32 (3.3)
	7.2 The skilled birth attendants and support staff have appropriate competence and skills to meet all the requirements	Increase professionalism, empathy, kindness and politeness	53 (13.5)	54 (5.6)
	7.3 Managerial and clinical leadership responsible for developing and implementing policies and fosters an environment that supports staff in quality improvement	Enable health managers to correctly shape new policies and rules in order to improve quality of care and enable staff to work more efficiently	5 (1.3)	5 (0.5)
Standard 8: appropriate physical environment, with adequate water, sanitation and energy supplies, medicines, supplies and equipment for routine maternal and newborn care and management of complications	8.1 Water, energy, sanitation, hand hygiene and waste disposal facilities are functional, reliable, safe and sufficient	Improve bathrooms and showers (eg, improve number and comfort; bathroom available for each room, and not only in the corridor)	72 (18.4)	72 (7.5)
		Perform a complete renovation of the maternity ward	30 (7.7)	30 (3)
		Improve the lightening of the post-delivery rooms	11 (2.8)	11 (1.1)
		Reduce noises and disturbance sources in the ward, especially at night	23 (5.9)	23 (2.4)
		Improve cleanliness	21 (5.4)	21 (2.2)
	8.3 An adequate stock of medicines, supplies and equipment is available for routine care and management of complications	Improve rooms equipment and personal health products by providing curtains between beds/changing tables/breast pumps /nursing chairs/sanitary napkins/soap/birthing bed/WIFI/disposal of waste/glasses/handles/tights with graduated compression/hangers)	35 (8.9)	40 (4.1)
Extra	Not included in WHO Standards	Improve privacy by decreasing number of women per room	29 (7.4)	29 (3.0)
		Consider adapting visiting areas	23 (5.9)	23 (2.4)
		Improve quality of meals provided including more attention to different diets needs (i.e. more differentiated food, bigger portions/different schedule/attention for allergies or intolerances)	19 (4.8)	19 (2.0)
		Improve case-clustering (ie, rooms assigned to women in the same condition, avoid putting women with different conditions (such as labours vs abortion) in the same room	14 (3.6)	14 (1.4)
		Reconsider rooming- in (ie, allow the possibility of get support from the nurses in taking care for the newborn, when needed -eg, when the mothers need to take a shower-, without strict rooming 24/24 h)	13 (3.3)	13 (1.3)
		Increase quantity of beverage provided for every woman	10 (2.6)	10 (1.0)
		Improve air conditioning use and allow possibility to open the windows (currently blocked as a suicide preventive measure)	6 (1.5)	5 (0.6)

Other frequent women’s requests were improving rooms equipment (8.9%) and increasing availability of hospital staff at any time when needed (8.2%).

Overall 114 women’s requests in this area were not related to any WHO standard, with the most frequent being: decreasing the number of patients per room (7.4% of women); creating separated areas for visitors (5.9%); improving quality of meals (4.8%); avoid case mixing in the same room, such as separating women with involuntary termination of pregnancy from those in labour (3.6%), and reducing strict rooming-in 24/24 h to allow the mother to rest or to shower (3.3%).

#### Comments without specific suggestions

Among the 89 comments without any specific or practical suggestion, 62 (15.8% of total women) were of appreciation, 18 (4.5%) were negative complaints, and 9 (2.2%) were unclear.

Among the comments of appreciation, about half (46.2%) were general appreciations to the whole service, one third (28.3%) were praising staff professionality, and the remaining were divided in equal parts among positive remarks related to the kindness of staff, and the structure of the ward. For example, one mother wrote: *“I met competent and very sensitive staff in both delivery and post-partum departments”.* Another made this comment: *“I felt really understood by all the staff and would recommend the structure to friends*”. While a third added *“I always felt respected in my dignity and even during the visits I was respected”.*

## Discussion

This study showed that collecting women’s suggestions on how to improve QMNC at hospital level in Italy was feasible and extremely relevant, highlighting critical inputs on aspects of care that should be improved in the opinion of service-users. Most of mothers provided more than one inputs and requests for improving the QMNC. This is the first study in Italy documenting views of women, after giving birth in a hospital, on how to improve the QMNC: we were able to identify three previous research reporting on the women’s experience of care during childbirth in Italy, but these did not include practical suggestions, as expressed by women, on how to improve it [25, 29, 30]. Additionally, this is the first study utilising the WHO Standards [19] as framework for the thematic analysis on women’s suggestions on how to improve the QMNC, and may serve as model for future research. Strengths of the study include that had a relatively large sample size, when compared overall to existing literature [20, 21].

Findings of this study are in line with existing evidences on women’s requests on QMNC, as reported by other investigations. The most frequent women’s request emerged in this study, ie, increase presence of a companion (made by about one third of women in our study) has been largely documented, together with the fear of being alone, in previous systematic reviews [20, 31]. Key aspects of “experience of care”, such as a request for privacy and confidentiality, effective communication and information, respect, empathy and continuity of care have been also widely documented [20, 31]. Other themes relevant to the “resources” domain, such as the need for a safe and supportive environment, and the expectation for health professionals to be skilled, competent, sensitive and kind, in the delicate moment of childbirth, have emerged as key themes in studies conducted both in low-income and high-income countries [20, 31]. Taken together this literature calls for more investments in establishing routine systems for collecting patients’ suggestions on how to improve QMNC, as already recommended by WHO and others [19, 32]. Data collected should be used in practice for planning intervention to improve QMNC (19.32). This pilot study may be used as a model for future projects, aiming at improving women participation into health care planning.

Interestingly, in this study only a minority of mothers (11.5%) made suggestions related to the domain of “provision of care”, which includes all technical aspects of evidence-based care (such as mode of delivery, type of practices experienced). Exiting similar studies conducted in high-income countries [19, 22] seem to confirm that women tend to make few suggestions related to medical practices. For example, in an Australian study [22], women reported as unique expectation related to “provision of care” the desire of a vaginal birth (when compared to operative delivery or caesarean section), and other important aspects (eg, induction of labour, episiotomy etc) were not mentioned at all. The observation that in our study as well as in others [16] few mothers made suggestions related to the “provision of care” may have multiple explanations, including: lack of knowledge among mothers on “technical aspects” of care; cultural factors including different types of stereotypes (eg, tendency to low autonomy in these domains); low importance attributed by mothers to these aspects when compared to others (eg, newborn well-being); overall good practices with low need for improving the QMNC. Each of these factors may play a different role in a different contest. More studies should document factors affection women’s opinion on “provision of care” in different settings.

This study identified, among overall women’s requests, 16.4% themes currently not included in the WHO Standards, such as a request for more privacy, and for more flexible rooming in (ie, not strictly 24/24 h) to allow the mother to rest or shower. These findings should be interpreted based on the characteristics of the setting and in the light of other women’s suggestions - such as the request for higher presence of a companion, and better comfort in the rooms, suggesting that the problem is not rooming-in *per se*, but rather the lack of other adequate support systems for the mother in the immediate post-delivery-. Currently, there is little experience on the use of the WHO Standards [19], no standardized data collection tool for high-income countries, nor pre-defined data sources. Our results, in line with existing literature [11, 32] call for further research in this area. If confirmed by other studies, consideration should be given on whether to include the additional themes emerged in this study as important for women, among the WHO Standards.

We acknowledge that this study was conducted in one single facility in Italy, and as such findings are not generalizable to other Italian hospitals. Previous studies reported clear differences in practices (such as caesarean section rate) as well as in the experience of care among different geographical regions in Italy, and even among nearby facilities in the same region [6, 29]. For example, it is possible that the maternal and newborn outcomes influenced indirectly the perception of the QMNC with Halo effect, ie, the behavior, usually unconscious, of using evaluations based on things unrelated, to make judgments about something or someone [33, 34].

Additionally, we acknowledge that this study, due to lack on interpreter, did not to capture views of migrant women unable of talking the local language (Italian). Recent studies pointed out how the need of migrant women may focus on specific topics, such as: the need for information on how to access health services, the availability of trained interpreters, and developing capacities health care providers on how to respond to the health needs of women with different backgrounds, in a culturally appropriate way [35, 36]. Similarly, women younger than 18 years were not included in the study, which therefore does not represent the views of adolescent mothers.

Finally, we acknowledge that the data collection tool (open questions to be filled on a voluntary basis) and the timing of administration (immediate post-partum) may have affected results. For example, some answers suggested that mothers may have much more to say, and this would deserve different data collection methods (eg. focus group). Additionally, soon after childbirth mothers may be more concentrated on the joy of having a baby than on the quality of care received. A Halo effect, with negative experiences being temporarily overshadowed in the immediate post-delivery by the excitement and joy of the moment, and later looming as time goes and memories become more realistic, has been previously described among women after delivery [33]. Additionally, although there were no major differences between mothers who provided their views and those who did not (Supplementary Table 2), we cannot exclude a selection bias. For example, it is possible that mothers who provided their views were those with more to say. However, it is also possible that women’s’ response rate was affected by other factors, such as individual psychological traits (eg, willingness to contribute, trust in the institution); contingency (eg, availability of time) or other factors. The ideal timing, tools and data collection methods for these types of studies have not been establish yet. Further research is needed to explore how mothers’ views may vary over time, depending on the timing and types of questions asked, and maternal and newborn health outcomes. In general, more studies should be conducted to further explore mothers’ suggestions and requests on how to improve QMNC in different regions in Italy, as well as in other countries.

Most importantly, data emergency from this and from similar studies should be used to improve QMNC. Efforts should be made for establishing routine systems for monitoring patients experience of care, and for ensuring that data collected are used for improving the quality of health services, as recommended by WHO and others [19, 32]. Still there is little evidence exploring the drivers of poor experience if care and even fewer studies documenting interventions to prevent it. As highlighted also by others [32], although studies and measurement remain important, we need to move beyond collecting data, and drive efforts for increasing accountability and for tracking and achieving change.

## Conclusion

Collecting the women’s views on how to improve the QMNC after hospital delivery highlighted critical inputs on aspects of care that should be improved in the opinion of service-users. More investments should be made for establishing routine systems for monitoring patients experience of care. Data collected should be used un practice to improve health service quality. WHO Standards may be further optimized by adding items emerging as relevant for women in high-income countries.

## Supplementary information

**Additional file 1 Table S1.** Standards for Reporting Qualitative Research (SRQR) Checklist. **Table S2.** Comparison with missing cases.

## Data Availability

The datasets used and/or analysed during the current study are available from the corresponding author on reasonable request.

## Abbreviations

QMNC: Quality of Maternal and Neonatal Care
SDG: Sustainable Development Goals
WHO: World Health Organization
PPH: Post-Partum Haemorrhage
GL: Guidelines
